# The Medical Annual

**Published:** 1889-03

**Authors:** 


*The Medical Annual.* 1889. Bristol: JOHN WRIGHT & CO.

We have pleasure in calling attention to and in commending this useful work. Its special features are the alphabetical arrangement of matter; the therapeutical synopses and reports; the very full bibliography at the end of each article; and last, but not least, the fact that the authors of the abstracts have modestly kept themselves and their opinions in the background, and have let the works quoted speak for themselves. There are one or two noteworthy exceptions, and these are mostly commendable. We would specially refer to the article by John W. Taylor, on the Diseases of Women in relation to dress, in which there is much suggestive and novel matter.

